# Endocarditis Caused by *Actinobaculum schaalii*, Austria

**DOI:** 10.3201/eid1607.100349

**Published:** 2010-07

**Authors:** Martin Hoenigl, Eva Leitner, Thomas Valentin, Gernot Zarfel, Helmut J.F. Salzer, Robert Krause, Andrea J. Grisold, Martin Hoenigl, Eva Leitner, Thomas Valentin, Gernot Zarfel, Helmut J.F. Salzer, Robert Krause, Andrea J. Grisold

**Affiliations:** Author affiliation: Medical University of Graz, Graz, Austria; Author affiliation: Medical University of Graz, Graz, Austria

**Keywords:** Actinobaculum sp., endocarditis, bacteremia, Actinobaculum schaalii, bacteria, Austria, letter

**To the Editor:** In May 2009, a 52-year-old man was hospitalized with middle cerebral artery stroke and fever of unknown origin. He had a complicated medical history of middle cerebral artery stroke and mechanical valve replacement of the aortic valve 2 years earlier and gastric-duodenal angiodysplasia. Two months before the most recent hospitalization, he had been hospitalized because of fever and anemia; blood cultures were positive; Gram stain identified coryneform rods that did not grow in culture. Antimicrobial drug therapy with levofloxacin (400 mg 1×/d) was initiated, and the patient was discharged.

At the most recent admission, laboratory testing showed a leukocyte count of 5.92 × 10^3^ cells/μL, with 81% neutrophils, 7% lymphocytes, and 9% monocytes; thrombocyte count was 338 × 10^3^ cells/μL. C-reactive protein level was 62.6 mg/L (reference value <8 mg/L). Basic serum and urine chemical profiles and urine culture were unremarkable. Empiric antimicrobial drug therapy with piperacillin–tazobactam (4.5 g 3×/d) was initiated and discontinued after 5 days because of clinical improvement. The next day, the patient’s condition deteriorated, C-reactive protein level increased from 15 mg/L to 32 mg/L, and blood was collected for culture on the day after piperacillin-tazobactam discontinuation and the next 2 days. After 4 days of incubation, bacterial growth was detected in 1 aerobic and 3 anaerobic samples. Gram stain showed positive coryneform rods. Within 48–72 hours, the isolate yielded growth on blood, chocolate, and Schaedler agar; colonies were 1–2 mm in diameter and gray. The specificity of the organism was unsatisfactory with the system we used (API Coryne system; bioMérieux, Craponne, France) (Table).

**Table:** Comparison of isolated Actinobaculum schaalii with related human pathogens reported in the literature

Characteristic	Isolate that caused endocarditis	Reaction of (reference)*				
		*A. schaalii* (*1**,**2*)	*Actinobaculum massiliae* (*3*)	*Actinobaculum urinale* (*4*)	*Arcanobacterium pyogenes*	*Actinomyces turicensis* (*1**,**2*)
	*A. schaalii*†					
Catalase reaction	–	–	–	–	–	–
β-hemolysis on sheep blood agar	–	–/w‡	–	w	+	w
Nitrate reduction	–	–	–	–	–	–
Pyrazinamidase	–	V	+	–	–	–
Pyrrolidonyl arylamidase	+	+	–	–	+	–
Alkaline phosphatase	+	–§	–	–	V	–
β-glucuroniadase	–	–	–	+	+	–
β-galactosidase	–	–	–	–	+	–
α-glucosidase	+	+	+	–	+	+
N-acetyl-β-glucosaminidase	–	–	–	–	V	–
Esculin hydrolysis	–	–§	–	–	–	–
Urease activity	–	–	–	+	–	–
Gelatin hydrolysis	–	–	–	–	+	–
Acid from						
Glucose	–	V	+	+	+	+
Ribose	+	+	+	+	+	+
Xylose	+	V	+	–	+	+
Mannitol	–	–	–	–	–	–
Maltose	+	+	+	+	+	V
Lactose	–	–	–	–	+	–
Sucrose	+	V	–	+	V	+
Glycogen	–	–	+	–	V	–

*API Coryne system (bioMérieux, Craponne, France) profile for our isolate was compared with those described in the references (*Actinobaculum schaalii* [14 strains], *A. massiliae* [1 strain], *A. urinale* [1 strain], *A. turicensis* [43 strains]) and those in the manufacturer´s database for *Arcanobacterium pyogenes*. +, >90% of strains positive; –, >90% strains negative; V, variable; w, weak. †In API Coryne, the strain gave the profile number 4110621 (unacceptable profile because of lack of specificity). ‡*A. schaalii* was described as nonhemolytic for 5 patients (*1*) and as showing weak β-hemolysis only after 2–5 d in 9 cases (*2*). §Reported as positive for 1 of 14 strains.

A 16S rRNA gene analysis was performed by using eubacterial universal primers. Subsequently, a BLAST search (www.ncbi.nlm.nih.gov/BLAST) of the partial 16S rRNA gene sequence (730 bp) was performed by using the taxonomy browser of the National Center for Biotechnology Information (www.ncbi.nlm.nih.gov). Homology of 99.7% (728/730 bp) was detected for *Actinobaculum schaalii*. The isolate was deposited in GenBank under accession no. GQ355962. MICs were obtained for various antimicrobial drugs, including amoxicillin–clavulanic acid (0.25 mg/L), piperacillin–tazobactam (0.125 mg/L), and levofloxacin (1 mg/L).

Infectious disease specialists were consulted. On physical examination, the patient exhibited Janeway lesions on hands and feet and a temperature of 38.4°C. Transesophageal echocardiogram showed filiform vegetation on the aortic valve, which was not consistent with echocardiographic major criteria. According to the modified Duke criteria (*5*), the patient’s condition fulfilled 1 major clinical criterion (at least 2 positive cultures of blood samples collected 12 hours apart) and 3 minor clinical criteria (prosthetic aortic valve, temperature >38°C, Janeway lesions). Accordingly, definite infective prosthetic valve endocarditis was diagnosed. Intravenous antimicrobial drug therapy with piperacillin-tazobactam (4.5 g 3×/d) was initiated, followed by oral therapy with amoxicillin–clavulanate acid (1 g 3×/d) for 8 weeks. Because a repeated transesophageal echocardiogram 10 days after initiation of antimicrobial drug therapy showed no infective endocarditis, heart surgeons declined to replace the prosthetic valve. The patient’s condition improved, and 2 weeks later he was discharged in good clinical condition.

Four species within the genus *Actinobaculum* have been described: *A. massiliae* (causing urinary tract infection [UTI] and superficial skin infection), *A. urinale* (isolated from human urine), *A. suis*, and *A. schaalii* (*1**,**3**,**4**,**6*). *A. schaalii*, which is difficult to identify by culture, has been reported to cause UTI in elderly patients with underlying urologic conditions; a few studies have reported subsequent urosepsis, abscess formations, and osteomyelitis (*1**,**3**,**6**–**9*). Recently, Bank et al. (*7*) reported development of a TaqMan real-time quantitative PCR for *A. schaalii* and consecutive detection of the organism in 22% of 252 routine urine samples of patients >60 years of age (*8*). Those findings suggest that *A. schaalii* is a common undetected pathogen, especially in elderly patients with unexplained chronic UTI.

We report infective endocarditis caused by *A. schaalii*. To our knowledge, infective endocarditis caused by *Actinobaculum* spp. has not been reported. However, several reports have documented endocarditis caused by *Arcanobacterium* spp. and *Actinomyces* spp., which are phylogenetically related to *Actinobaculum* spp (*10*).

Characteristics of the patient reported here differed from those of patients in previous reports. He had no underlying urologic condition and could not recall any symptoms usually associated with UTI during the year before hospital admission. Urine culture remained negative for *Actinobaculum* spp. despite prolonged incubation for 5 days on chocolate agar in an atmosphere of 5% CO_2_ and on Schaedler agar under anaerobic conditions.

This report highlights the usefulness of the recent development of a specific real-time PCR by Bank et al. (*7*), which may prove effective not only for patients typically at risk for *A. schaalii* but also for patients with a wider spectrum of infection. More studies are needed to identify the real prevalence of disease caused by this difficult-to-cultivate organism because it may occur in many other groups of patients.
